# Delayed diagnosis of Visual Snow Syndrome due to misdiagnosis as conversion disorder : a rare case report

**DOI:** 10.1192/j.eurpsy.2025.1810

**Published:** 2025-08-26

**Authors:** L. S. Chaibi, S. Hallit, S. Majoul, M. Cheour, S. Ellini, M. Bouchendira, F. Fekih-Romdhane

**Affiliations:** 1 Department of Psychiatry, Razi Hospital, Mannouba, Tunisia; 2 School of Medicine and Medical Sciences, Holy Spirit University of Kaslik, Jounieh, Lebanon; 3 Department of Neurology, Razi Hospital; 4 Department of Psychiatry, University Tunis El Manar, Mannouba, Tunisia

## Abstract

**Introduction:**

Visual snow syndrome (VSS) is a rare condition that presents as a form continuous visual disturbance that occupies the entire visual field, described as tiny flickering dots. Although VSS might be expressed in patients with migraine as visual aura, persistent VSS has been accepted as a distinct clinical entity. Symptoms of VSS commonly emerge in late adolescence and early adulthood, but it can affect people of any age. In the literature,a few cases of childhood VSS have been reported.The peculiarity of this condition is that it remains rare and challenging to diagnose.

**Objectives:**

Here, we report an uncommon case of VSS in a Tunisian female adolescent aged 17 years, that has been misdiagnosed for years as Conversion Disorder/Functional Neurological Disorder.

**Methods:**

A clinical history, neurological assessment and visual tests were performed, leading to the diagnosis VSS in a misdiagnosed 17-year-old Tunisian female.

**Results:**

A nine-year-old girl with no prior medical history started experiencing symptoms of vision of white lines in a net and abnormal image persistence throughout her entire visual field. The patient initially consulted an ophthalmologist, then a neurologist. The patient’s visual field testing, retinal imaging, and eye examination were all within the normal range. Electroencephalography, cerebrospinal fluid analysis, and brain MRI revealed nothing unusual. The psychogenic origin was initially considered, and the patient was referred to a psychiatrist. The diagnosis of functional neurological disorder was established. The patient underwent cognitive behavioral therapy with no improvement.Over the next years, the patient consulted several psychiatrists, but her visual symptoms did not improve. Gradually, her symptoms worsened, with a significant impact on her academic performance. New symptoms such as entopic phenomena appeared, with floaters, sparkling dots, and luminous flashes associated with palinopsia and photophobia. A tinnitus was also associated. At the age of 17, and given the therapeutic impasse, her treating psychiatrist concluded that she needed to be referred back to neurology and ophthalmology. She had to undergo all the complementary tests again, which once more came back without abnormalities. The neurologist retained the diagnosis of VSS. The patient was put on anti-epileptic and migraine medications, which were ineffective. Food supplements were also prescribed to reduce stress. The patient underwent psychotherapy with partial recovery. Being correctly diagnosed helped her better cope with her symptoms. Her academic results substantially improved.

**Conclusions:**

Despite its rarity, VSS should be taken into consideration in any patient whose vision has been continuously pixelated. More research is required to pinpoint the actual cause of this condition. Beyond its difficult diagnosis, the pathogenesis is uncertain, which the treatment plan problematic as well.

**Disclosure of Interest:**

None Declared

